# Supplementary material for: Acceptability and playability of an organization training videogame for young adolescents with ADHD: The development of ATHEMOS
Source: PLOS Digit Health. 2023 Nov 13;2(11):e0000374. doi: 10.1371/journal.pdig.0000374 (PMC10642850; doi:10.1371/journal.pdig.0000374)
Supplement: Attachment — Submitted filename: Response to Reviewers.docx [file pdig.0000374.s001.docx]

Response to Reviewers

Please note that line numbers were drastically altered after moving large sections in the manuscript. So, the line designations below refer to the changes as they appear in the new, marked-up revised manuscript. We believe this will make it easy for reviewers to identify our edits.

Reviewer #1:

1. Introduction. In the sentence "School-based behavior interventions can alleviate some of the academic and social impairments of ADHD, but there are persistent implementation challenges that require interventions to be designed and tested specifically for those settings." I lack the references to the evidence which point this out.

- We added a reference to support this claim (Lyon et al) on line 26.

2. I would move the game development and description to the methods section.
   - We agree that this works better and made that change (see pp 5-7).
3. Materials and methods. In the participants section, lines 202-215 belongs to the procedure description and not the description of the participants. And I would move the demographics to the start of the results section and include how you analyzed this information in the statistical analyses section.
   - We understand the reviewer’s concerns but believe the best solution is to change the subheading to “Participant Recruitment” to better capture what we present in this section (line 115). Similarly, we do not believe the participants’ demographics are a “result” of the study and kept that information in the method section. We did, however, move some additional recruitment information from later in the paper into this section to achieve parallel construction with the focus group details immediately above (see lines 141-146). We hope the reviewers will agree that these changes improve this section of the paper.
3. Results. Include all abbreviations used in tables in the Note part of the table.
   - Only Table 2 includes abbreviations, and we define both BF (Bayes Factor) and CI (credible interval) in the table note. The other abbreviations are common statistical notations (e.g., *M*, *SD*, *n*) that we believe readers of PLOS Digital Health will be familiar with.
4. Discussion. More reference to how your results compare to previous evidence in the field should be mentioned.
   - This is a challenging request because the game we are examining represents a nearly unique approach to game-based intervention. And when new educational games are developed, user acceptability is rarely assessed by comparing the new product to purely recreational games, as we have done here. The only comparable study we are aware of is already cited (Espinoza-Curiel et al., 2020), so we added more detail to emphasize how rarely this comparison is made (line 469).

Reviewer #2: This reviewer is not a child psychiatrist or paediatrician, and is not an expert in ADHD, but is aware of the harm caused by amphetamine-like medications, and given the reflexive prescription of those medications, any scalable non-pharmacological intervention is of considerable interest. However, the premise that such a large proportion of children have that condition would seem likely to undermine the validity of an intervention that appears to be trying to attract the interest of children said to have that condition in order to train them to overcome symptoms.

- We’re not sure we understand the reviewer’s concern, but we updated the ADHD prevalence statistics at the start of the manuscript using a recent study (Bitsko et al., 2022, lines 16-18). That source suggests that 4.3 to 4.9 million public school students in the United States have ADHD, which we believe underscores the need for easy-to-implement, low-cost interventions in school settings.

The paper does not include outcome data, or a detailed account of how a study to evaluate the intervention might be conducted, including recruitment, inclusion and exclusion criteria, outcome measurement, or a rationale for how it might work.

- We clarified the focus of this study in the “Current Study” subsection by explaining the critical importance of treatment acceptability (lines 95-99). Clinical outcomes are a separate question; it is critical to first establish that this novel intervention is acceptable to the target population. So, in this article we limit our focus to that concern. We also believe that the background section describing the Challenging Horizons Program provides the rationale for why we think the game-based intervention using those same elements might ultimately work and expanded on previous findings to help convey that point (lines 28-69).

Reviewer #3: MAJOR ISSUES

The main issue is that the paper does not describe exactly the aims.

- We revised the “Current Study” subsection to address this concern and believe that this change, combined with the serial list of our two aims, now conveys this information effectively (lines 93-110).

There is a very long section about the development of the computer program, but no data regarding the perception and efficacy of ATHEMOS in clinical practice. It is reported in the limitations, that “research is needed to demonstrate… real-world outcomes”, but reading the manuscript is not clear, before the real end of itself.

- Reviewer #2 voiced a similar concern, and in response to both we clarified the focus of this study in the “Current Study” subsection by explaining the critical importance of treatment acceptability—the focus of this study (specifically, lines 95-99). See our response above for more details.

The reports of the eight focus group are not reported, as well the suggestion of the pilot sample (except the results of a psychometric scale, GUESS). I suggest the authors to change the title, to be more focused on what the reader should expect from the manuscript, as well in the abstract and in the introduction.

- The GUESS data are our focus group results. As explained in the manuscript, plans for additional qualitative measures (e.g., transcribed interviews) were cancelled due to social distancing policies implemented during the COVID-19 pandemic. Considering this reviewer’s feedback, we made wording changes in the focus group procedures section to emphasize that point (lines 312-313 and lines 318-319). But the GUESS is a particularly strong, norm-referenced instrument, with multiple subscales that speak directly to our interests as game developers. Both the focus group and pilot study results are shown in Table 2, with highlights discussed in the body of the paper.
- As to the title, we believe that “acceptability and playability” accurately describes the GUESS and satisfaction data we analyze in the paper. As to the abstract and introduction, we made changes that we believe better announce our aims (e.g., Current Study section; last sentence of Abstract).

Abstract: the sentence “implications of these findings” should be removed from the abstract. It is more useful to include what implications are expected, since most of readers read the abstract before the study itself, and this would provide a more appealing interest in the reader.

- We deleted that sentence. Instead, we end the abstract with a new sentence briefly summarizing our conclusions (and better prefacing our aims).

In the abstract no mention of the Athemos is reported.

- Good catch. We added “ATHEMOS” to the abstract.

Line 18: a reference is needed.

- We added a recent reference to support our claim about the prevalence of ADHD (Bitsko et al., 2022, lines 16-18) and updated those statistics accordingly.

Line 28-34: to the general audience, the CHP could be something not known. A more extensively explanation of the program is needed.

- We expanded the description of the CHP. See lines 31-37, and lines 45-46.

Furthermore, the series of limitations of the CHP should be more contextualized. An explanation of its costs should be provided. The difference between CHP and CHP-C should be more detailed.

- The largest cost the CHP is staffing, which we tried to highlight in our expanded description of the program (lines 32-34). To distinguish the CHP-C, we added a brief description of school consultation (specifically lines 45-46).

Line 187: provide the reference number of the ethical center.

- We added the IRB approval numbers at our respective institutions at the top of the Method section. Hopefully this satisfies the reviewer’s concern (lines 112-114).

Line 212: the sample size of the pilot study should be reported.

- The size of the treatment arm of the pilot study is reported (*n* = 16) (see lines 139 and 382). If the reviewer is referring to the control group that was not included in the present study (an additional 15 students), we would prefer to leave it unmentioned because it might cause confusion for readers.

Table 1: explain “biracial”.

- Parents were asked if they identified their child as “biracial” and interpreted that term however they wished. We don’t believe there is any advantage in defining that term further or offering additional details beyond mentioning that demographic data were collected from parents using standard forms (lines 149-150).

Line 243: the authors reported that the serious game was “revised as needed”. However no mention of what changes were made is present. The authors should describe the process of collecting feedback and the change made to the program.

- We added a summary of the changes we made to the game in the focus groups procedures section (lines 318-319).

Line 258: the teacher mentor were trained in the used of the program? How? When?

- We expanded the description of the CHP-C to better differentiate it from the CHP (lines 45-46), and added a brief description of the consultative support that teacher mentors received (lines 348-349).

Line 277: a reference is needed regarding the GUESS scale.

- A reference is provided at the top and near the bottom of this paragraph, so it should be clear to the reader that all information provided here is attributable to that source. We believe additional citations would be overkill.

MINOR ISSUES

GPA at line 45 is not defined.

- We deleted that parenthetical note to avoid confusion because it was unnecessary (line 53).

Line 210: correct the punctuation.

- Fixed—thank you for catching this.
