# Supplementary material for: Acceptability and playability of an organization training videogame for young adolescents with ADHD: The development of ATHEMOS
Source: PLOS Digit Health. 2023 Nov 13;2(11):e0000374. doi: 10.1371/journal.pdig.0000374 (PMC10642850; doi:10.1371/journal.pdig.0000374)
Supplement: Attachment — Submitted filename: Response to Reviewers_R1.docx [file pdig.0000374.s002.docx]

Response to Reviewers, PDIG-D-23-00108R1

Please note that our line references below are to the marked-up revision of the manuscript.

Journal Requirements:

1. Please review your reference list to ensure that it is complete and correct. If you have cited papers that have been retracted, please include the rationale for doing so in the manuscript text, or remove these references and replace them with relevant current references. Any changes to the reference list should be mentioned in the rebuttal letter that accompanies your revised manuscript. If you need to cite a retracted article, indicate the article's retracted status in the References list and also include a citation and full reference for the retraction notice.

We previously cited one source that was under review that has since been rejected. But we can safely remove that citation (line 91) without changing the relevant sentence because we are making a claim about our understanding of the literature (i.e., “To our knowledge ATHEMOS is the first serious game in English that trains organization and planning skills for students with ADHD, paired with school-based intervention to achieve skill transfer”). We checked all other references, and we believe everything is in order.

2. Please send a completed 'Competing Interests' statement, including any COIs declared by your co-authors. If you have no competing interests to declare, please state "The authors have declared that no competing interests exist". Otherwise please declare all competing interests beginning with the statement "I have read the journal's policy and the authors of this manuscript have the following competing interests:"

We will make these changes in the upload system too, but here is the full statement:

I have read the journal’s policy and the authors of this manuscript have the following competing interests:

“The first two authors, BKS and SWE, are exploring options to potentially redesign and commercialize the videogame and intervention package described herein, but there were no active commercial interests at the time of submission.”

3. Please amend your detailed Financial Disclosure statement. This is published with the article. It must therefore be completed in full sentences and contain the exact wording you wish to be published.

a. State the initials, alongside each funding source, of each author to receive each grant.
b. If any authors received a salary from any of your funders, please state which authors and which funders.

If you did not receive any funding for this study, please simply state: “The authors received no specific funding for this work.”

The research described in this article was supported by a grant (R324A180219) from the US Department of Education Institute of Education Sciences (IES) to East Carolina University (BKS) and Ohio University (SWE). The opinions expressed herein are those of the authors and do not represent the views of IES or the U.S. Department of Education. IES had no role in the research methodology, data collection or analysis, or preparation of this manuscript.

*Please note that our funding source, IES, requests that the financial disclosures are worded in this manner.*

4. Figures 1-5 contains screenshots. We are not permitted to publish these under our CC-BY 4.0 license; websites are usually intellectual property and are copyrighted. This includes peripheral graphics of the web browser such as the buttons. We ask that you please remove or replace it.

We’d like to clarify that the videogame depicted in those images was developed specifically for this project, and nothing in the images is proprietary to secondary software or hardware programs.  Per communication with Sarah Mayo on 6/27, our understanding is that these images will work.

Additional Editor Comments (if provided):

The research was interesting. Please consider the following minor issues in your revision.

1-      Please make sure that you have added appropriate keywords after the abstract.

We believe our current list of keywords makes sense but are open to suggestions if there is a concern. Those were added immediately following the abstract.

2-      In the introduction section, please remove subheadings, integrate them, and present a full introduction section.

In our revisions, we added a new subheading “Introduction” (line 15) and removed all other subheadings in the introduction section. Hopefully this satisfies the concern.

3-      In the methods section, please start with the “study design”.

We changed the first subheading in the Method section from “Participant Recruitment” to “Study Design” (line 115).

4-      Table 1 can be moved to the results section.

We prefer to leave Table 1 where it is because it reports participant demographics, which are not the “results” of our study. In our view, readers will want to know about our sample early in the method section, even before we start describing the game we designed and tested, and we don’t want to potentially confuse readers into believing that the table represents our results.

5-      In the methods section, if your research composed of several phases, you can separate them to help the readers to know what happened at each phase, who the participants were, what the research tool was, etc.

We carefully considered this feedback. There were two “phases” as we switched from focus groups to the pilot study, and that distinction is already delineated on pages 7-8 (lines 248-284). If we rename these subheadings to Phase 1 and 2, or refer to them in that manner, our concern is that readers will get confused. So, although we appreciate the feedback, we believe the current naming strategy is the most straightforward.

6-      All details of the game development and Figures can be moved to the results section to make it more informative. If you separate phases of your research in the methods section, the results section can also be reorganized to show the results of each phase separately.

We’re not sure this would improve the manuscript because the development of the game was our method—if we moved that information to the results, the method section would not accurately reflect our process. Again, if the editor agrees, we would like to keep the content in the method and results sections where they are, even though the method is much longer than the results. Perhaps in the publication phase, the figures might be spread out between the method and results section to balance the page counts (?), but we believe the callouts make most sense where they are currently placed.

7-      In the discussion section, please start with a summary of findings and then support the results with the existing literature and similar studies.

We made several changes to the discussion section to better summarize our findings and to tie our findings back to the existing literature. A summary sentence is now provided on lines 398-399. Then, in the next paragraph (lines 413-419), we relate our findings to two articles discussed previously in the manuscript describing the differing interest in videogames between boys and girls. We added two citations to that same literature on line 469, and then added another citation [25] to research discussing the potential for games to teach intermediate skills (e.g., problem-solving) that can have real-world benefits. Note that for this latter citation, we added an earlier mention on lines 209-210 in our description of the minigames to ensure this idea was familiar to readers prior to the discussion. We believe these changes tie our results back to the existing literature effectively, without overwhelming the reader.
